# Virus-Like Particles Activate Type I Interferon Pathways to Facilitate Post-Exposure Protection against Ebola Virus Infection

**DOI:** 10.1371/journal.pone.0118345

**Published:** 2015-02-26

**Authors:** Natarajan Ayithan, Steven B. Bradfute, Scott M. Anthony, Kelly S. Stuthman, Sina Bavari, Mike Bray, Keiko Ozato

**Affiliations:** 1 Program in Genomics of Differentiation, National Institute of Child Health and Human Development, National Institutes of Health, Bethesda, MD, 20892, United States of America; 2 United States Army Medical Institute of Infectious Diseases, Fort Detrick, MD, United States of America; 3 Integrated Research Facility, National Institute of Allergy and Infectious Diseases, National Institute of Health, Fort Detrick, MD, United States of America; George Mason University, UNITED STATES

## Abstract

Ebola virus (EBOV) causes a severe hemorrhagic disease with high fatality. Virus-like particles (VLPs) are a promising vaccine candidate against EBOV. We recently showed that VLPs protect mice from lethal EBOV infection when given before or after viral infection. To elucidate pathways through which VLPs confer post-exposure protection, we investigated the role of type I interferon (IFN) signaling. We found that VLPs lead to accelerated induction of IFN stimulated genes (ISGs) in liver and spleen of wild type mice, but not in *Ifnar^-/-^* mice. Accordingly, EBOV infected *Ifnar^-/-^* mice, unlike wild type mice succumbed to death even after VLP treatment. The ISGs induced in wild type mice included anti-viral proteins and negative feedback factors known to restrict viral replication and excessive inflammatory responses. Importantly, proinflammatory cytokine/chemokine expression was much higher in WT mice without VLPs than mice treated with VLPs. In EBOV infected *Ifnar^-/-^* mice, however, uninhibited viral replication and elevated proinflammatory factor expression ensued, irrespective of VLP treatment, supporting the view that type I IFN signaling helps to limit viral replication and attenuate inflammatory responses. Further analyses showed that VLP protection requires the transcription factor, IRF8 known to amplify type I IFN signaling in dendritic cells and macrophages, the probable sites of initial EBOV infection. Together, this study indicates that VLPs afford post-exposure protection by promoting expeditious initiation of type I IFN signaling in the host.

## Introduction

Ebola viruses (EBOVs) are enveloped, negative-sense RNA filoviruses that can cause a severe hemorrhagic fever in humans and non-human primates (NHPs) [1, 2]. Mouse-adapted EBOV causes similar acute disease in mice, offering a useful animal model to study EBOV infection [3, 4]. EBOV infection is characterized by rapid viral replication and dysregulated innate and adaptive immune responses. The disease follows profound suppression of type I IFN signaling and a contrasting excess inflammation that leads to mucosal hemorrhages and multi-organ failure resembling septic shock syndrome [5, 6].

Virally encoded anti-IFN proteins, VP24 and VP35 play major roles in EBOV virulence [7, 8]. VP35 blocks type I IFN induction in dendritic cells (DCs) and macrophages, and acts as a virulence factor necessary for a recombinant virus to attain infectivity in the host [9–12]. VP24, on the other hand, blocks IFN signaling by interfering with IFN activated JAK/STAT pathways [7].

Lines of evidence support the critical importance of type I IFN signaling in providing resistance against EBOV infection; mice deficient in *Stat1*, a transcription factor required for IFN induction, or *Ifnar1*, encoding the membrane receptor for type I IFNs, are susceptible to wild type Zaire EBOV, against which wild type mice are resistant [13–15]. A study of Sudan EBOV infection in humans showed that IFNα levels are significantly higher in surviving patients than those with fatal EBOV infection, who had higher levels of proinflammatory cytokines/chemokines such as IL-6, and MIP-1β [16, 17]. High IFNα production is reported to correlate with increased resistance against EBOV in mice as well [18]. Administration of recombinant IFNα or IFNβ confers delayed time-to-death in NHPs [19, 20]. Furthermore, IFNα, used as an adjunct therapy for monoclonal antibody treatment, is shown to enhance protection in NHPs [21]. EBOV infection remains a potential threat to public health, which is compounded with the lack of effective prevention or treatment. To overcome this problem, various vaccine candidates have been developed, including various DNA constructs, recombinant viruses, VLPs, as well as treatment with anti-sense siRNA [22–24].

VLPs are subunit-based vaccines, extensively studied for a variety of infectious pathogens [25, 26]. VLPs prepared from EBOV and other filoviruses are composed of the matrix protein (VP40), glycoprotein (GP), and at times nucleoprotein (NP) and represent a potentially promising candidate for EBOV vaccine. EBOV VLPs have been shown to confer protection upon rodents and NHPs when given prior to infection [27–29]. In the accompanying paper, we show that post-exposure administration of trivalent VLPs protects mice from lethal EBOV infection, further crediting the potential of VLPs as a possible vaccine [30]. In that study, we show that VLP protection requires macrophages, dendritic cells (DCs) as well as B and either CD4 or CD8 T lymphocytes, indicating that both innate and adaptive immunity are involved in conferring protection. The aim of this study was to further investigate molecular bases of post-exposure protection by VLPs. Based on our previous report that VLPs stimulate type I IFN expression in DCs and macrophages, in vitro, we focused on the role of type I IFN signaling, and found that post-exposure VLP treatment leads to accelerated activation of IFN signaling, resulting in early induction of ISGs. Significantly, VLP stimulated ISG induction coincided with the attenuation of proinflammatory cytokine surge in EBOV infected mice. The reduced inflammatory responses was attributed to activation of type I IFN signaling, since VLP treated *Ifnar* ^-/-^ mice were unable to inhibit not only viral replication but proinflammatory responses, and succumbed to death. Our results indicate that early type I IFN response is a major mechanism that contributes to VLP mediated protection against EBOV infection.

## Materials and Methods

### Mice and ethics statement

*Ifnar* ^*-/-*^, *Ifnar* ^*+/+*^ mice of BALB/c background and *Irf8* ^*-/-*^ and *Irf8* ^*+/+*^ mice of C57BL/6 background were bred in the NICHD animal facility and transferred to the facility of the United States Army Medical Research Institute of Infectious Diseases (USAMRIID) for EBOV infection studies. Research was conducted in compliance with the Animal Welfare Act and other federal statutes and regulations relating to animals and experiments involving animals and adheres to principles stated in the guide for the Care and Use of Laboratory Animals, National Research Council, 1996. The facility where this research was conducted is fully accredited by the Association for Assessment and Accreditation of Laboratory Animal Care International. The IACUC committee approving this protocol is the United States Army Medical Research Institute of Infectious Diseases (USAMRIID) IACUC. Animals were monitored at least once daily and their status was evaluated according to an intervention score sheet approved by USAMRIID IACUC. Monitoring increased to three times daily if the animals were given a score of three or four. Euthanization was by CO_2_ inhalation followed by confirmatory cervical dislocation. Analgesics and anesthetics were not used in this study and animals were euthanized for humane purposes if they reached a score of five or more, which would be indicated if the animals exhibited ruffled fur, weakness, unresponsiveness, and/or difficulty walking. Otherwise, animals were euthanized at the end of the study.

### VLPs and EBOV infection

VLPs were composed of EBOV GP, NP and VP40 and were generated in mammalian 293T cells as reported previously [31]. VLP preparations used in this study contained <0.03 endotoxin U/mg. Mice were infected with ~1000 pfu (~3,000 LD_50_) of mouse-adapted EBOV via intraperitoneal (i.p.) route [32]. Mice were injected with VLPs (50 μg) diluted in PBS through i.p. 24 h after EBOV infection. Morbidity and mortality of EBOV infected mice were monitored twice daily for up to 14 days.

### Quantitative real-time PCR

Total RNA from liver and spleen of EBOV infected mice were extracted by TRIzol method (Invitrogen) and cDNA was synthesized from 1 μg total RNA by Superscript II reverse transcriptase (Invitrogen). qPCR amplification was done with 3 ng cDNA in 5 μl SYBR Green PCR master mix (Applied Biosystems) with 3 μM of both reverse and forward primers used in the ABI prism 7500 Sequence Detection System (Applied Biosystems). mRNA of expression of indicated genes were analyzed as described in detail elsewhere [33]. The primer pairs were used for EBOV GP, 5’-TGGGCTGAAAACTGCTACAATC-3’ and 5’- CTTTGTGCACATACCGGCAC-3’; NLRP3, 5’-TGCTCTTCACTGCTATCAAGCCCT-3’ and 5’-ACAAGCCTTTGCTCCAGACCCTAT-3’. All other gene primer sequences were followed from the previous publications [10, 33]. Transcript levels were normalized with Hprt, and expressed as relative expression. Statistical analysis was carried out by Excel software using two-tail paired Student’s *t* test. Data represent the mean of at least three independent assay ± SEM. A *p* value < 0.05 was considered significant.

## Results

### Lack of VLP mediated post-exposure protection in *Ifnar1* ^*-/-*^ mice against EBOV infection

**Fig 1:**
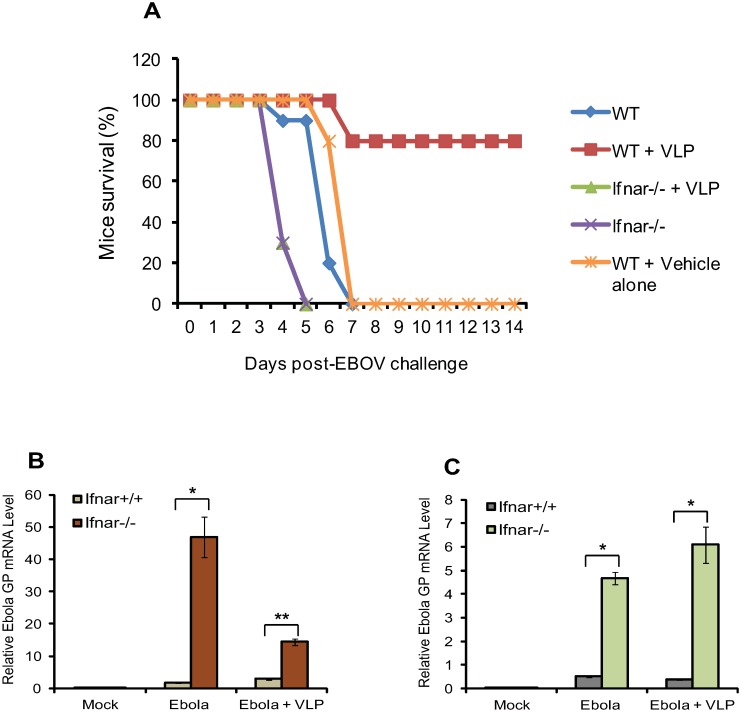
IFNAR is required for VLP post-exposure protection against EBOV. (A) *Ifnar* ^*-/-*^ and *Ifnar* ^*+/+*^ (WT) mice (n = 10 mice/group) were infected i.p. with ~1000 PFU ma EBOV, followed 24 h later by injection i.p. with 50 μg of EBOV VLPs. One group of *Ifnar* ^*+/+*^ (WT) mice infected with EBOV without VLP served as control. Mortality is expressed as percent survival of each group on indicated days. Results are a representative of three independent experiments, which gave very similar outcomes. QRT-PCR detection of EBOV GP mRNA level on day 2 post-EBOV infection with or without VLPs from liver (B) and spleen (C) of WT or *Ifnar* ^*-/-*^ mice. GP transcripts were normalized by Hprt and values represent the mean ± SEM of duplicate samples from three independent experiments. Asterisk denotes significant differences compared to WT controls (*p ≤ 0.05, **p ≤ 0.01).

To assess the role of type I IFN signaling in VLP-mediated protection against EBOV infection, we tested *Ifnar1* ^*-/-*^ mice for protection by VLPs. In Fig. 1A, wild type (WT) and *Ifnar1* ^*-/-*^ mice (both BALB/c background, n = 10) were injected with 50 μg of VLPs 24h after infection by the mouse adapted (ma) EBOV, and the morbidity and mortality were checked daily for the subsequent 14 days. WT mice without VLP injection all died between day 5 and day 7, whereas 80% of mice that received VLPs survived after EBOV infection, confirming that VLPs protect mice even when they were given post-infection. In contrast, *Ifnar* ^*-/-*^ mice that received VLPs all died before or at day 5 as those without VLP injection (Fig. 1A). These results are in agreement with previous report on early death of ma-EBOV infected *Ifnar* ^*-/-*^ mice [15].

EBOVs are thought to initially infect DCs and macrophages in liver and spleen, making these tissues the major sites of EBOV replication in the mouse, although the virus infects many other organs later [3, 34]. To ascertain whether VLPs inhibit viral replication, we measured EBOV glycoprotein (GP) mRNA expression in liver and spleen from WT and *Ifnar* ^*-/-*^ mice with or without VLP administration. QRT-PCR analysis in Fig. 1B and 1C, found that levels of GP mRNA rose sharply in *Ifnar* ^*-/-*^ mice on day 2 of infection when GP mRNA was still at background in WT mice. *Ifnar* ^*-/-*^ mice that received VLPs also expressed considerable amounts of GP mRNA, although levels varied between liver and spleen in the VLP treated group. Thus, EBOV appeared to replicate faster and to a greater extent in *Ifnar* ^*-/-*^ mice than WT mice. It should be noted here that in WT mice, GP mRNA levels began to increase rapidly after day 2, peaking on day 3 to day 5, and that VLP injection inhibited GP mRNA expression by more than half (S1 Fig.).

These results are in line with the results that *Ifnar* ^*-/-*^ and *Stat1* ^*-/-*^ mice are more susceptible to EBOV infection, suggesting the possibility that VLP mediated protection is linked to the activation of type I IFN signaling [13–15]. However, VLP injection may not have prevented EBOV pathogenesis in *Ifnar* ^-/-^ mice, possibly because the disease manifests more severely in these mice than in WT mice. On the other hand, it has been recently shown that Adenovirus based vaccine can protect *Ifnar* ^*-/-*^ mice from lethal EVOB infection presumably through antibody responses, which indicates that *Ifnar* ^*-/-*^ mice are not universally vulnerable, and anti-EBOV resistance can be attained in some cases [35].

### VLP treatment accelerates induction of anti-viral and negative feedback ISGs in EBOV infected mice

**Fig 2:**
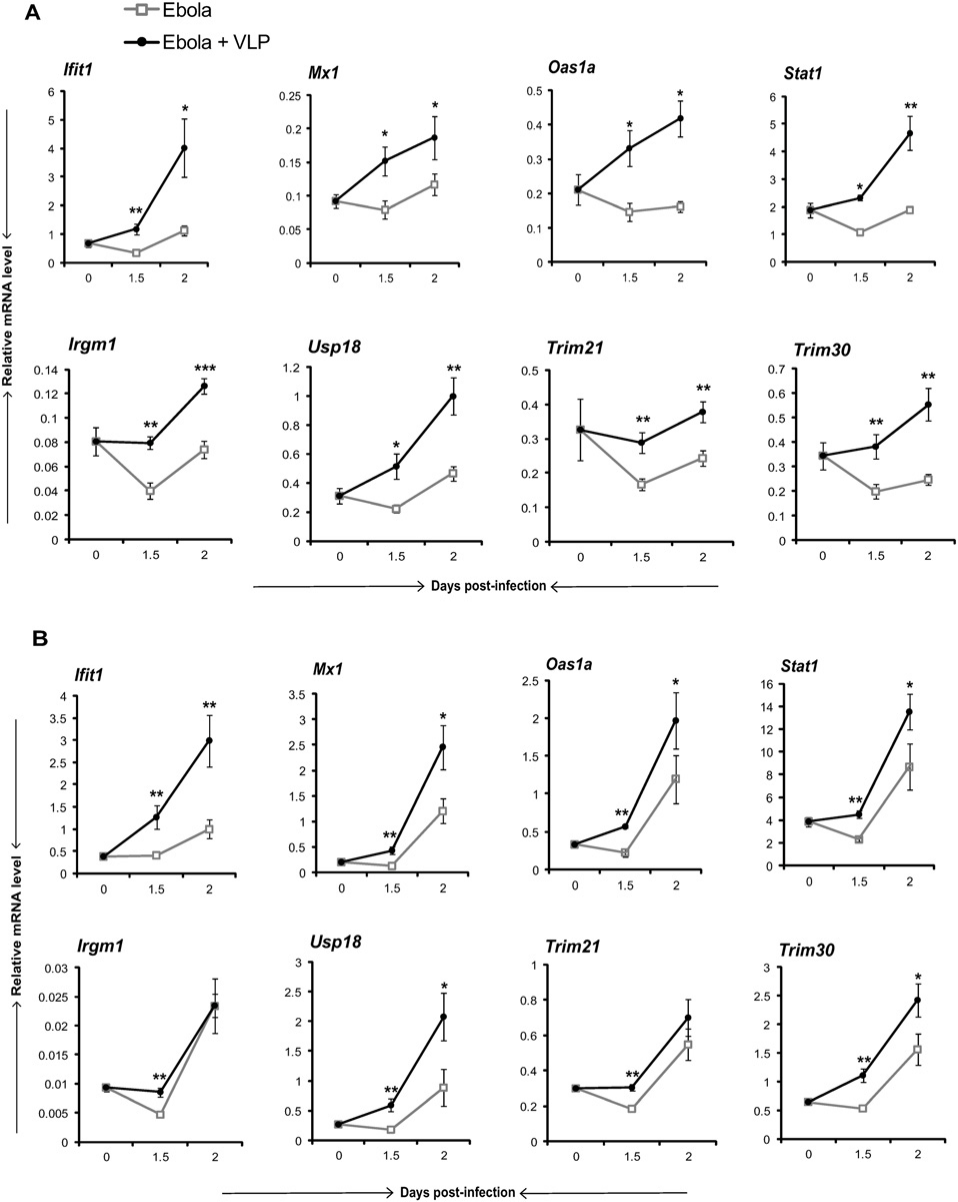
Accelerated ISG induction in VLP treated mice. WT mice were injected with VLPs 24 h post-EBOV challenge (n = 5/group) and transcripts of indicated ISGs in liver (A) and spleen (B) were measured on days 0, 1.5 and 2 post-infection. Ifit1, Mx1, Oas1a and Stat1 represent ISGs with anti-viral activity, while Irgm1, Usp18, Trim21 and Trim30 are ISGs with negative regulatory activities. Values represent the mean of duplicate samples from three to five independent experiments ± SEM. *** denotes *P* ≤ 0.001, ** *P* ≤ 0.01 and *P ≤ 0.05.

We recently reported that EBOV VLPs activate type I IFN transcription in DCs and macrophages in vitro, leading to induction of many ISGs in these cells [33]. Here we asked whether VLPs stimulate ISG induction in vivo. WT mice were infected with ma EBOV and received VLPs 24 h later, and induction of ISG mRNAs was tested on days 1.5 and 2. ISGs encoding anti-viral proteins were first examined, as they may provide early protection against EBOV infection. Upper panels in Fig. 2A and 2B compare induction of anti-viral ISGs, Ifit1, Mx1, Oas1a and Stat1 with or without VLP injection in liver and spleen. In this early stage, levels of these ISGs were consistently higher in the VLP-injected groups than those without VLPs. At later stages of infection, however, the situation reversed, in that mice without VLPs had higher levels of ISGs, as seen on day 3 (S1 Fig. for complete kinetics). These results indicate that VLP administration accelerated type I IFN and ISG induction, which presumably provide early anti-viral activity, not afforded without VLPs.

We next tested whether VLPs induce other ISGs, particularly those with negative regulatory activities. This question was of interest to us, since mice that did not receive VLPs expressed higher levels of proinflammatory cytokines and chemokines, which raised the possibility that IFN signaling exerts negative regulatory activity towards proinflammatory responses, perhaps by controlling NF-κB activation [36]. Shown in the lower panels in Fig. 2A and 2B is induction of IRGM1, USP18, TRIM21 and TRIM30. IRGM1 is an IFN inducible GTPase that inhibits LPS induced endotoxin shock in mice [37]. USP18 is an ISG15 deconjugating factor that negatively regulates TLR signaling and resultant cytokine induction [38]. Trim21 and Trim30 are members of the Tripartite motif family that downregulate TLR induced inflammatory responses [39–42]. Expression of these ISGs was also higher in the VLP injected group than that without VLPs both in liver and spleen. Similar to anti-viral ISGs, expression of these negative regulatory factors changed at the later stage (S1 Fig). These data indicate that VLPs accelerate induction of anti-viral and negative regulatory ISGs, which may help suppress EBOV’s anti-IFN antagonism (See Discussion).

To confirm that VLP induction of ISG is dependent on type I IFN signaling, we next tested ISG induction in *Ifnar* ^*-/-*^ mice. As expected, none of the ISGs tested in Fig. 2 were induced in *Ifnar* ^*-/-*^ mice after VLP treatment or EBOV infection (S2 Fig).

### VLPs lower expression of proinflammatory cytokines in EBOV infected mice

**Fig 3:**
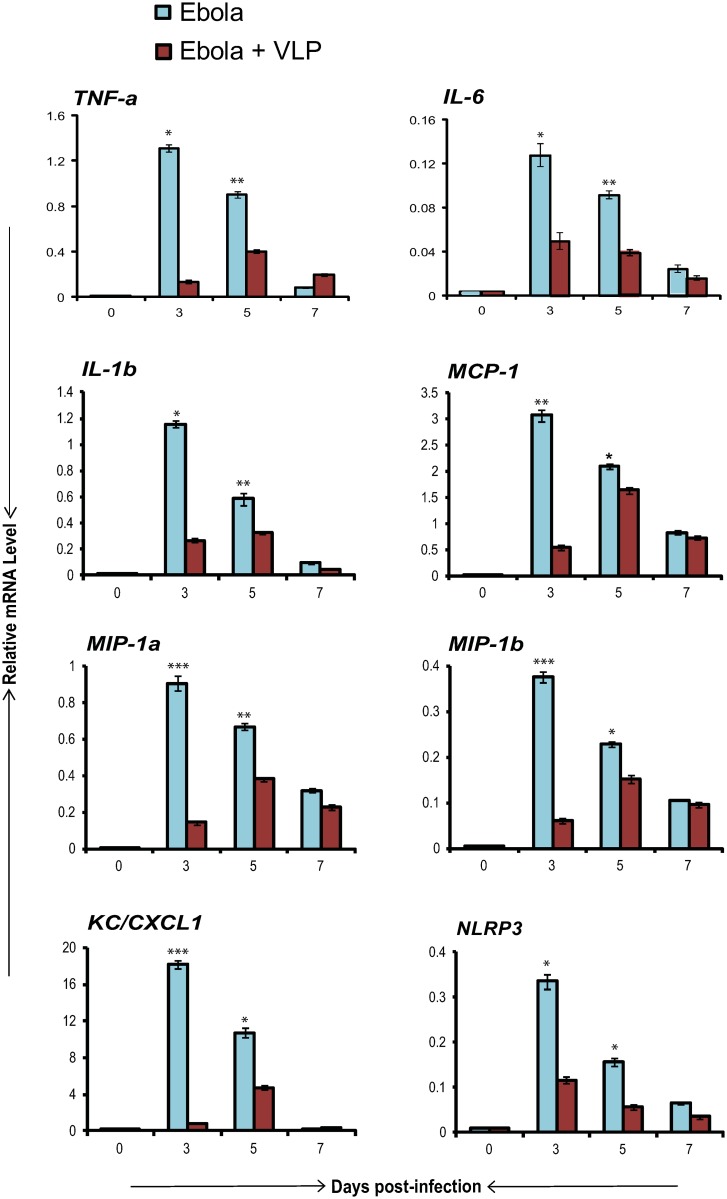
VLPs attenuate proinflammatory cytokine induction in EBOV infected mice. RNA from liver of WT mice (n = 5/group) infected with EBOV with or without VLP treatment was tested for expression of proinflammatory cytokines (TNFα, IL-6 and IL-1β), chemokines (MCP-1, MIP-1α, MIP-1β and KC (CXCL1)) and Inflammasome gene NLRP3 on indicated days post-infection. Values represent the average of duplicate samples from three independent experiments ± SEM. *p ≤ 0.05, **p ≤ 0.01, ***p ≤ 0.001 indicates statistical significance.

EBOV pathophysiology such as severe hemorrhagic symptoms and tissue damage is thought to be associated with dysregulated inflammatory cytokine production [2, 43]. Given that VLPs accelerated induction of negative regulatory ISGs, we next evaluated whether VLPs modulate expression of proinflammatory genes. In Fig. 3, expression of TNFα, IL-6 and IL-1β, chemokines such as MCP-1 (CCL2), MIP-1α (CCL3), MIP-1β (CCL4), KC (CXCL1) and inflammasome gene NLRP3 was measured in EBOV infected mice with or without VLPs. These genes were all strongly induced upon EBOV infection and peaked on day 3 with a gradual decline on days 5 and 7. In all cases, their expression was significantly attenuated in the VLP-treated group as compared to the group without VLPs. The difference was most dramatic in the early stage on day 3, where the expression was reduced at least by 50%. In agreement with these results, we noted that serum levels of some of these proinflammatory cytokines were higher in EBOV infected mice that were treated with VLPs as compared those without VLPs [30]. These results support the view that limiting superfluous inflammatory responses contribute to VLP mediated protection.

### Exacerbated induction of proinflammatory cytokines and chemokines in *Ifnar* ^*-/-*^ mice

**Fig 4:**
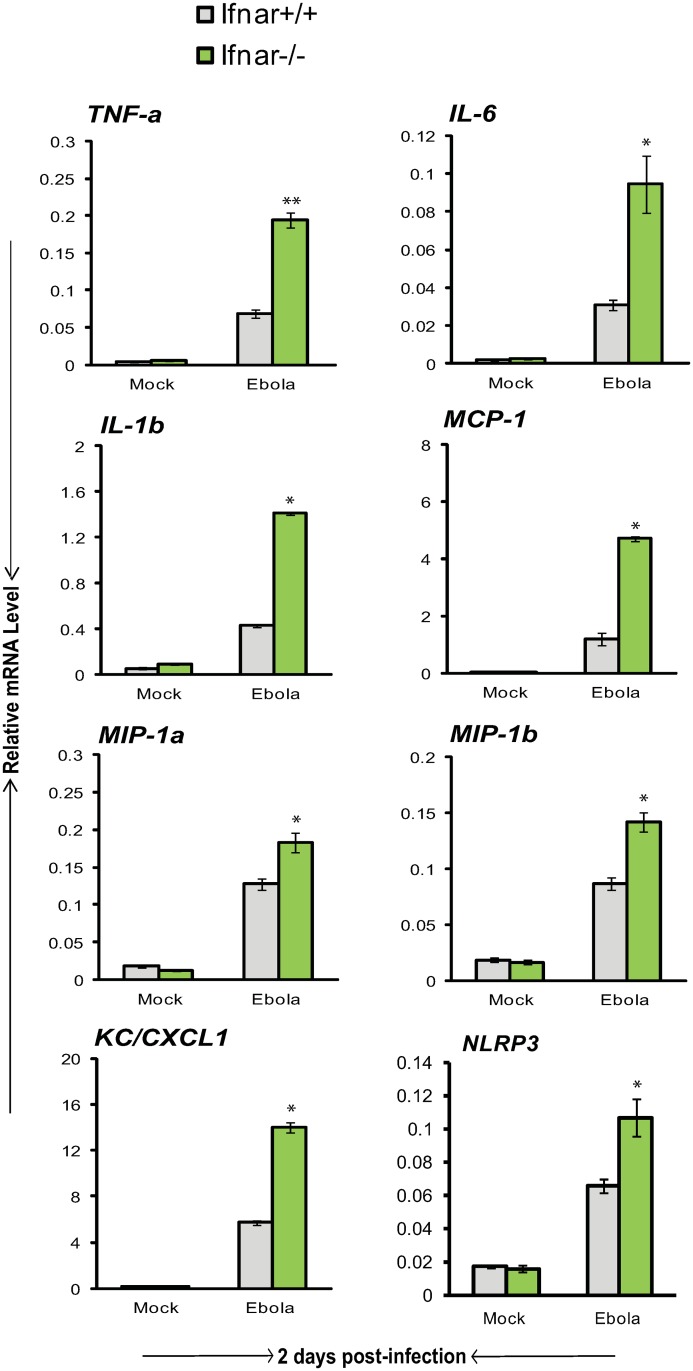
Heightened induction of proinflammatory cytokines and chemokines in EBOV infected *Ifnar* ^*-/-*^ mice. Liver RNA from *Ifnar* ^*+/+*^ and *Ifnar* ^*-/-*^ mice (n = 5/group) infected with EBOV was tested for expression of indicated cytokines or chemokines on day 2 post-infection. Values represent the mean of duplicate samples from three individual experiments ± SEM. Asterisks denote **P ≤ 0.01, *P ≤ 0.05.

Increasing evidence indicates that type I IFNs antagonize inflammatory responses in a variety of settings [44–46]. In light of the results that VLPs stimulate those ISGs known to suppress proinflammatory responses, it was of importance to determine whether type I IFN signaling by and of itself affects EBOV induction of proinflammatory cytokines and chemokines. Results in Fig. 4 and S3 Fig compare expression of the above proinflammatory factors in *Ifnar* ^*+/+*^ and *Ifnar* ^*-/-*^ mice infected with EBOV. All cytokines and chemokines tested were induced after EBOV infection in both strains. Importantly, their levels were much higher in *Ifnar* ^*-/-*^ mice than *Ifnar* ^*+/+*^ mice. These results indicate that type I IFN signaling downregulates EBOV stimulated induction of proinflammatory factors, possibly through ISGs with negative regulatory activities.

### *Ifnar* ^*-/-*^ macrophages elicit exaggerated proinflammatory cytokine responses following LPS and IFNβ signaling

**Fig 5:**
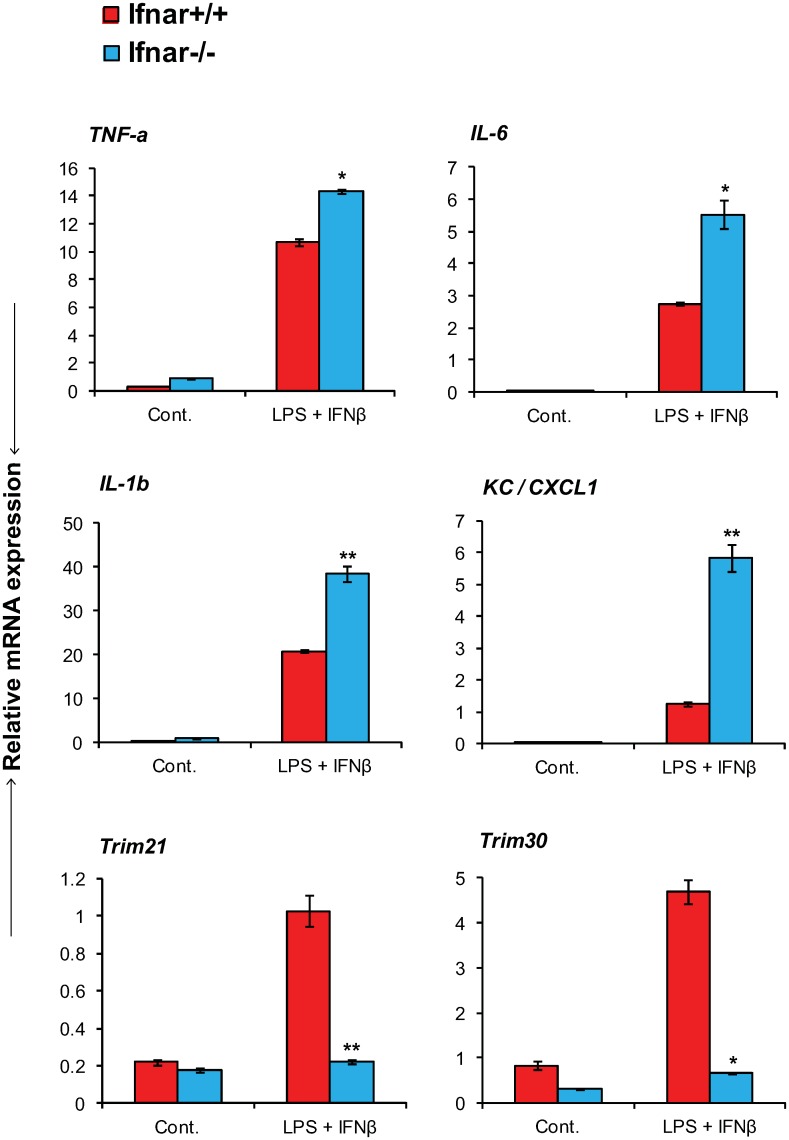
*Ifnar* ^*-/-*^ macrophages elicit exaggerated proinflammatory cytokine responses following LPS and IFNβ stimulation. Bone marrow-derived macrophages from *Ifnar* ^*+/+*^ and *Ifnar* ^*-/-*^ mice (1x10^6^ cells/well) were primed with IFNγ (100U/ml) followed by co-stimulation with LPS (200ng/ml) and IFNβ (100U/ml) for 3 h. Expression of indicated ISGs, proinflammatory cytokines and chemokines were assessed by qRT-PCR. Data represent the average of duplicate samples from three individual experiments ± SEM. Asterisks denote *p ≤ 0.05, **p ≤ 0.01.

The above results indicated that type I IFNs attenuate proinflammatory responses during EBOV infection. To explore whether type I IFNs have a similar activity in settings other than EBOV infection, we next tested LPS and IFNβ induced inflammatory responses in macrophages in vitro. LPS activates NF-κB mediated proinflammatory cytokine induction, which can result in endotoxin shock [36]. As shown in Fig. 5, combined treatment with LPS and IFNβ led to hyper induction of TNFα, IL-6, IL-1β and a chemokine KC in *Ifnar* ^*-/-*^ macrophages as compared to WT cells. LPS and IFNβ also induced negative feedback factors, Trim21 and Trim30, with much lower expression in *Ifnar* ^*-/-*^ cells than *Ifnar* ^*+/+*^ cells. These results support a model in which type I IFNs negatively regulate proinflammatory cytokine/chemokine responses at least in some situations. We found that MIP-1α, MIP-1β and MCP-1 were not hyperinduced in *Ifnar* ^*-/-*^ cells, suggesting that some proinflammatory genes are regulated not only by type I IFNs but other factors (data not shown). Alternatively, these differences may reflect variances between in vivo and in vitro conditions.

### VLP-mediated protection depends on the downstream transcription factor IRF8

**Fig 6:**
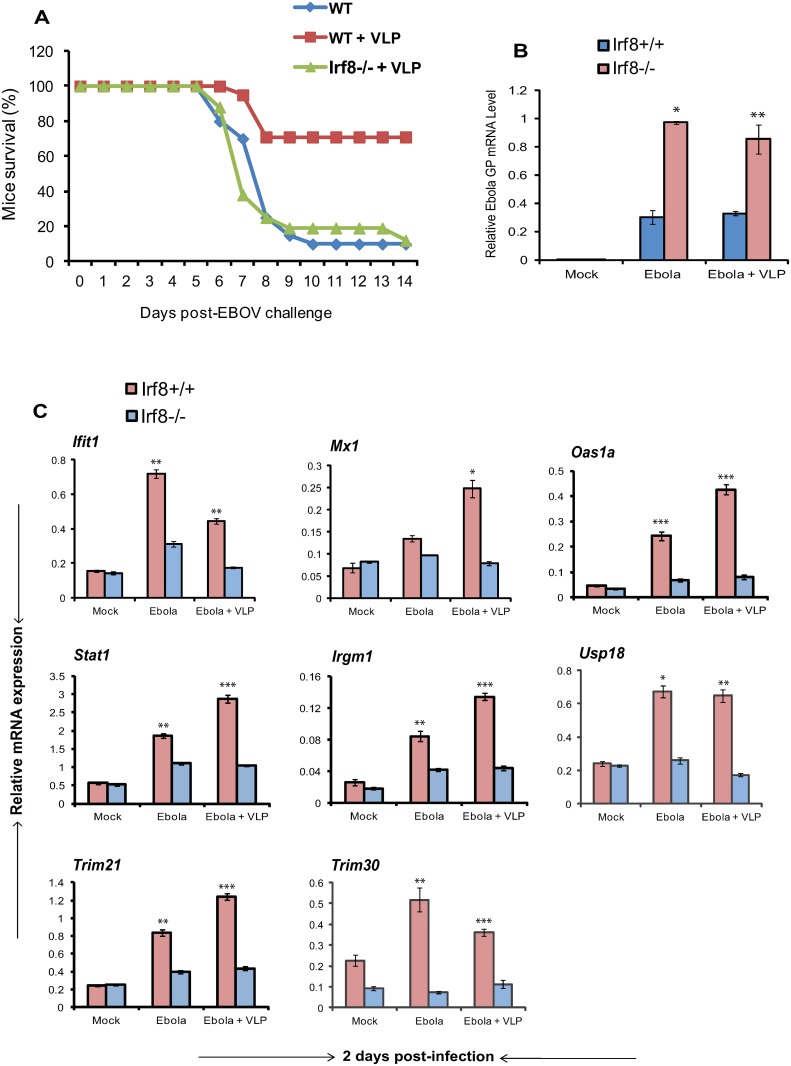
VLP post-exposure protection requires the transcription factor IRF8. (A) *Irf8* ^*+/+*^ (WT) and *Irf8* ^*-/-*^ mice (n = 5/group) were infected with EBOV followed by VLP treatment as in Fig. 1A. Mortality is expressed as the percentage survival. (B) EBOV GP levels in liver were tested by qRT-PCR on day2. (C) Expression of indicated ISGs in *Irf8* ^*+/+*^ and *Irf8* ^*-/-*^ liver was tested by qRT-PCR on day 2. Data represent the mean of duplicate samples ± SEM and a representative of three independent experiments. *** indicates *P* ≤ 0.001, ** *P* ≤ 0.01 and *P ≤ 0.05.

To further define pathways downstream of IFNAR activity, important for VLP protection, we directed our attention on IRF8, a transcription factor expressed in macrophages and DCs [47–49]. IRF8 is induced by IFNs and TLR ligands in a *Stat1* dependent manner, and plays a pivotal role in facilitating innate immune responses. Although IRF8 is not involved in initial triggering of type I IFN induction, it amplifies IFN transcription in DCs and macrophages [50]. IRF8 promotes induction of multiple anti-microbial factors and is required for innate resistance against a variety of pathogens [50–52]. IRF8 stimulates expression of MHC and costimulatory molecules to boost antigen presentation [48, 50]. We thus tested whether IRF8 disruption affects VLP-mediated protection against EBOV. Survival data in Fig. 6A show that approximately 80% of *Irf8* ^*-/-*^ mice that received VLPs died between day 6 and 8, which is nearly identical to the mortality curve of WT mice without VLPs. As expected, the majority of WT mice that received VLPs survived against EBOV infection. It is of note that *Ifnar* ^*-/-*^ mice died 1 to 2 days earlier than *Irf8* ^*-/-*^ mice, which may be attributed to the difference in the mouse background. Correlating with the lack of protection, Ebola GP mRNA levels were much higher in EBOV infected *Irf8* ^*-/-*^ mice than *Irf8* ^*+/+*^ mice with or without VLPs (Fig. 6B). We next examined whether induction of anti-viral and negative feedback ISGs is dependent on IRF8. Data in Fig. 6C illustrate that induction of these ISGs was very meager in *Irf8* ^*-/-*^ mice, in contrast to robust induction in *Irf8* ^*+/+*^ mice. Importantly, VLPs did not rescue ISG induction in *Irf8* ^*-/-*^ mice. Results were similar in liver and spleens (Fig. 6C and S4 Fig). These results indicate that VLPs, upon initial activation of type I IFN cascade, rely subsequently on the activation of downstream pathways represented by IRF8 to confer protection against EBOV.

## Discussion

To gain insight into the pathways through which VLPs confer resistance against EBOV infection, we investigated the role of type I IFN signaling in vivo and found that it significantly contributes to VLP-mediated protection. This conclusion is supported by the observation that post-exposure VLP treatment accelerated ISG induction in EBOV infected mice, leading to reduced viral replication and inflammatory gene expression. Further supporting the critical role of type I IFN signaling in the protection, VLPs did not induce ISGs in *Ifnar* ^*-/-*^ mice, and did not protect the mice from lethal EBOV infection. These results are consistent with the report that post-exposure IFNβ or IFNα treatment increases protection against EBOV infection in NHPs [1, 6, 15, 19].

It is likely that VLPs initially stimulated Type I IFN genes, which in turn led to early induction of ISGs. In line with this notion, we recently showed that exogenous VLPs stimulate transcription of IFNα and IFNβ in DCs and macrophages in vitro, an event coupled with immediate and robust ISG induction [33].

It may be reasonable to assume that *Ifnar* ^*-/-*^ mice were not protected by VLPs primarily because ISG induction was absent. However, *Ifnar* ^*-/-*^ mice may be susceptible to infection due to additional defects in innate immunity that are a secondary consequence of defective IFN signaling, which obliterates VLPs protection. Contouring this notion however, it is of note that *Ifnar* ^*-/-*^ mice can be protected against EBOV by an adenovirus-based vaccine, indicating that *Ifnar* ^*-/-*^ mice are not totally without defense [35]. Rather, it is possible that *Ifnar* ^*-/-*^ mice are not protected by VLPs that rely on ISG induction for protection, whereas they are protected by the adenovirus vaccine that depends on antibody response.

**Fig 7:**
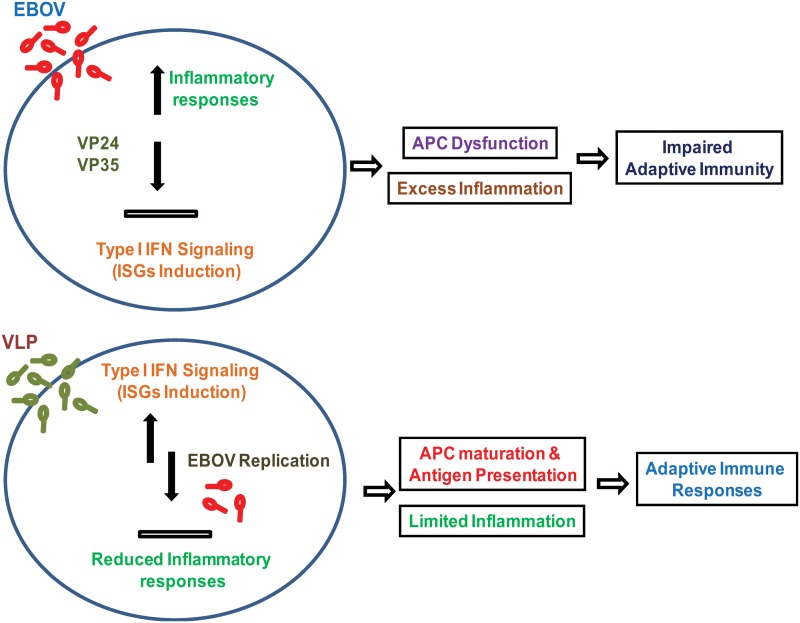
A model for VLP mediated post-exposure protection against EBOV infection. Top: EBOV infection in macrophages and DCs inhibits induction of type I IFNs and delays induction of ISGs via the mechanism of anti-IFN antagonism. Impaired type I IFN signaling delays induction of antiviral ISGs, and hinders maturation of macrophages and DCs and development of antigen presentation activity. It also inhibits prompt induction of negative regulatory ISGs that leads to exaggerated inflammatory responses. As a result, adaptive immunity is not adequately induced. Bottom: VLPs trigger timely induction of type I IFNs and ISGs by overcoming EBOV’s anti-IFN antagonism. This prompts rapid onset of anti-viral activity, simultaneously limiting exaggerated inflammatory responses and allowing maturation of antigen presentation function, which results in robust adaptive immune responses.

VLP-induced ISGs included anti-viral proteins known to inhibit replication of RNA viruses such as Ifit1, Mx1 and Oas1a, as well as negative feedback factors that curb excess inflammatory responses, such as Irgm1, Usp18, Trim21 and Trim30. Although the question of which anti-viral ISGs are effective in inhibiting EBOV replication awaits further research, it is anticipated that some of anti-viral ISGs induced by VLPs may interfere with EBOV life cycle [53]. What is the significance of accelerated IFN response in VLP mediated protection? Available evidence suggests that VLPs may overcome EBOV’s anti-IFN antagonism. The virally encoded VP24 and VP35 disable the entire IFN system in the host; while VP24 blocks the JAK/STAT pathway of IFN signaling, VP35, an EBOV virulence factor, inhibits type I IFN induction in many cell types [6, 7, 9, 11]. We previously showed that VP35 inhibits type I IFN induction in murine DCs by premature SUMOylation and inactivation of IRF7 [10]. It is thought that VP24 and VP35 have a decisive effect on the subsequent host resistance, since abated IFN signaling would impair proper innate immune responses, leading to deficiency in DC maturation, defective antigen presentation and aberrant inflammation. Compromised innate immunity would consequently undermine development of adaptive immunity [6] (See a model in Fig. 7).

It is remarkable that in the VLP treated mice, ISG induction began early within 1.5 to 2 days after EBOV infection (which was only 0.5 to 1 days after VLP treatment), when little to no ISG induction was seen in mice without VLPs. The delayed ISG induction in EBOV infected mice is reminiscent of the reports showing that influenza virus delays ISG induction in lung epithelial cells through NS1, an influenza anti-IFN protein that is linked to disease pathology [54, 55]. An influenza virus strain deficient in NS1 is shown to induce ISGs earlier than wild type virus, although the wild type strain does stimulate ISGs later on [54, 55]. Supporting the view that viral anti-IFN factors stall ISG induction, rather than completely abrogate the induction, we also observed ISG induction on Day 3 and later in mice without VLPs. It may be envisaged that VLPs trigger IFN activation early on, thereby eluding the activity of the EBOV anti-IFN proteins (a model in Fig. 7).

The most striking observation made in this study is the VLP-dependent suppression of proinflammatory responses. This suppression was a result of type I IFN signaling, as *Ifnar* ^*-/-*^ mice expressed higher levels of proinflammatory cytokines and chemokines, observed not only after EBOV infection but also by IFNβ and LPS stimulation. These results are in accordance with the growing recognition that type I IFNs are linked to attenuation of inflammatory responses [6, 44]. For example, Pinto et al., reported, in the West Nile Virus infection model that *Ifnar* ^*-/-*^ mice express excess proinflammatory cytokines, including those found in this study, as compared to WT mice, which correlated with increased disease pathology. In this system, the overt inflammatory responses were attributed to IFN signaling in macrophages and DCs [46].

Induction of proinflammatory cytokines and chemokines may be negatively regulated by IFN signaling through a series of negative feedback factors [37, 39–42, 56–58]. IRGM1, induced by IFN signaling restricts LPS induced endotoxin shock without limiting IFNβ expression [37]. TRIM21 and TRIM30 inhibit proinflammatory cytokine induction, at least in part by interfering with the NF-κB dependent arm of transcription [39–41]. In addition, these factors may act by post-transcriptional mechanisms, affecting inflammasome activation [42]. In this regard, Guarda et al. [45] reported that type I IFNs inhibit production of IL-1 by inhibiting activity of the NLRP1 and NLRP3 inflammasomes and by IL-10 induction. Thus, ISGs with negative regulatory activity may preferentially attenuate proinflammatory pathways, while sparing IFN induction pathways. Given our earlier observations that VP35 does not grossly affect NF-κB activation, while strongly inhibiting type I IFN activation, EBOV may promote proinflammatory pathways at least in part through VP35 [10].

Lastly, we show that the transcription factor IRF8 is required for VLP mediated post-exposure protection. Our results offer an added mechanistic insight into the pathways through which VLPs provide protection. IRF8 is expressed predominantly in macrophages and DCs, and helps to amplify type I IFN gene induction and boosts IFNs biological activities [51]. Given that macrophages and DCs are the putative early sites of EBOV infection, VLPs may exert a major impact on these cells to facilitate early innate immunity, in an IRF8 dependent manner.

In conclusion, VLPs confer post-exposure protection upon EBOV infected mice by rapidly inducing ISGs, thereby permitting timely establishment of anti-viral and anti-inflammatory states in the host. VLPs may act primarily by relieving EBOV’s antagonism against type I IFNs, resulting in reduced systemic inflammation and subsequent enhancement in acquired immune responses (a model in Fig. 7).

## Supporting Information

S1 Fig Time course of ISG induction in EBOV infected mice with or without VLP treatment. WT mice were injected with VLPs 24 h post-EBOV infection. Induction of EBOV GP levels in liver (A) and spleen (B) were tested for WT mice infected with EBOV with or without VLP treatment at indicated times (n = 5/group). Expression of indicated ISGs was also assessed by qRT-PCR from day 0 to day 7 in liver (C) and spleen (D). Data represent the mean of duplicate samples from more than three independent experiments ± SEM. *** denotes *P* ≤ 0.001, ** *P* ≤ 0.01 and *P ≤ 0.05.
(TIF)

S2 Fig VLP augment type I IFN-responsive genes via IFNAR-dependent manner. *Ifnar* ^*+/+*^ and *Ifnar* ^*-/-*^ mice (n = 5/group) were injected with VLP alone and expression of indicated ISGs and cytokine/chemokine in liver was measured on day 2 as shown in Fig. 3. Ebola infected mice served as positive control. Data represent the mean of duplicate samples from more than three independent experiments ± SEM. *** denotes *P* ≤ 0.001, ** *P* ≤ 0.01, *P ≤ 0.05. n.s., No significance.
(TIF)

S3 Fig Exacerbated induction of proinflammatory cytokines and chemokines in *Ifnar* ^*-/-*^ mice. RNA from spleen of *Ifnar* ^*+/+*^ and *Ifnar* ^*-/-*^ mice (n = 5/group) infected with EBOV was tested for expression of indicated cytokines or chemokines on day 2 post-infection. Data represent the mean of duplicate samples from three individual experiments ± SEM. Asterisks denote **P ≤ 0.01 and *P ≤ 0.05.
(TIF)

S4 Fig IRF8 is required for post-exposure protection by VLPs against EBOV infection. Relative mRNA expression of indicated ISGs in spleen on day 2 post-infection (n = 5/group) was analyzed as mentioned in Fig. 6C. Data are the mean of duplicate samples ± SEM and a representative of three independent experiments. *** indicates *P* ≤ 0.001, ** *P* ≤ 0.01 and *P ≤ 0.05.
(TIF)
