# Supplementary figures and images for: Virus-Like Particles Activate Type I Interferon Pathways to Facilitate Post-Exposure Protection against Ebola Virus Infection
Source: PLoS One. 2015 Feb 26;10(2):e0118345. doi: 10.1371/journal.pone.0118345 (PMC4342244; doi:10.1371/journal.pone.0118345)

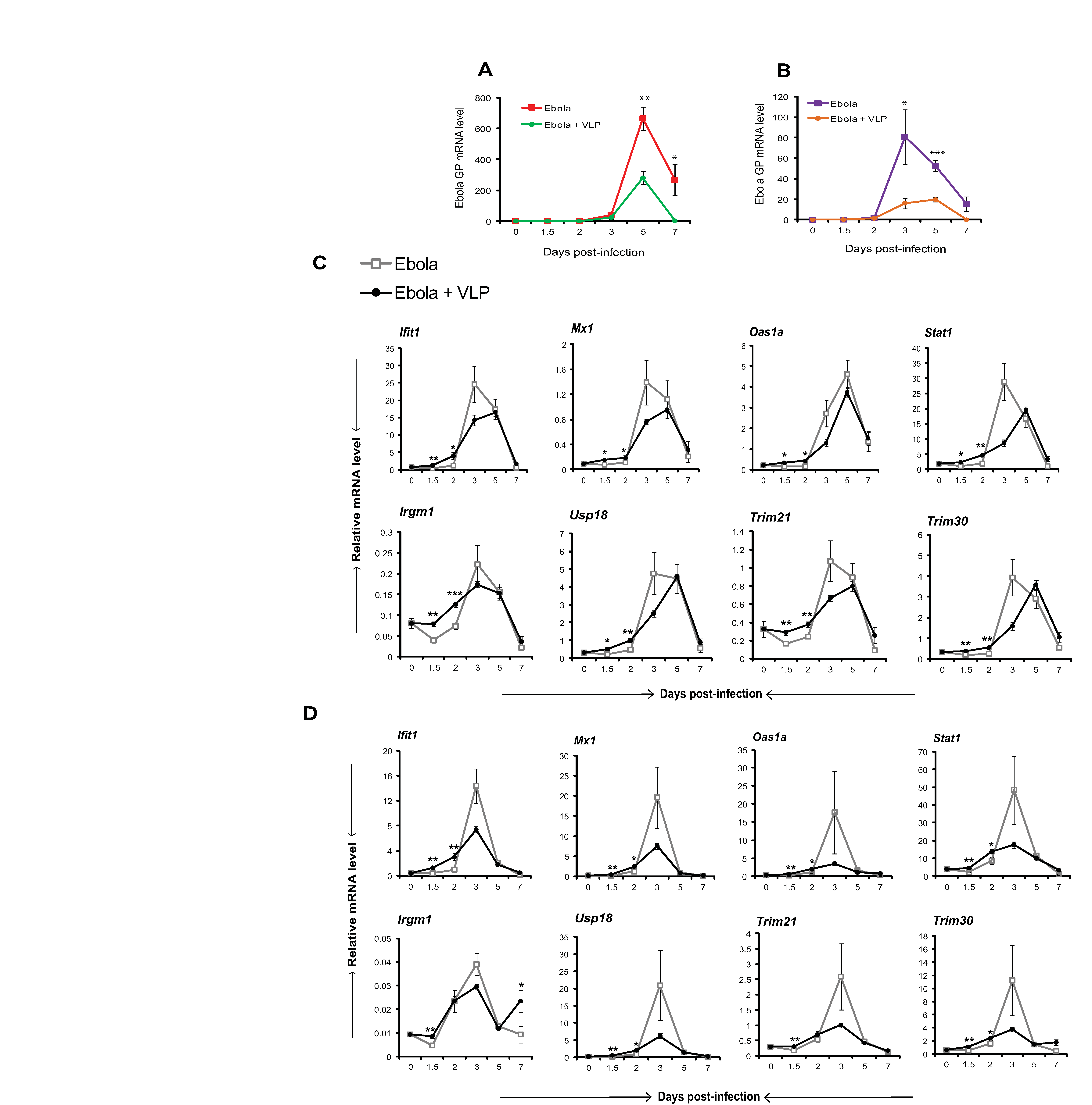

Supplement: S1 Fig — WT mice were injected with VLPs 24 h post-EBOV infection. Induction of EBOV GP levels in liver (A) and spleen (B) were tested for WT mice infected with EBOV with or without VLP treatment at indicated times (n = 5/group). Expression of indicated ISGs was also assessed by qRT-PCR from day 0 to day 7 in liver (C) and spleen (D). Data represent the mean of duplicate samples from more than three independent experiments ± SEM. *** denotes P ≤ 0.001, ** P ≤ 0.01 and *P ≤ 0.05. (TIF) [file pone.0118345.s001.tif]

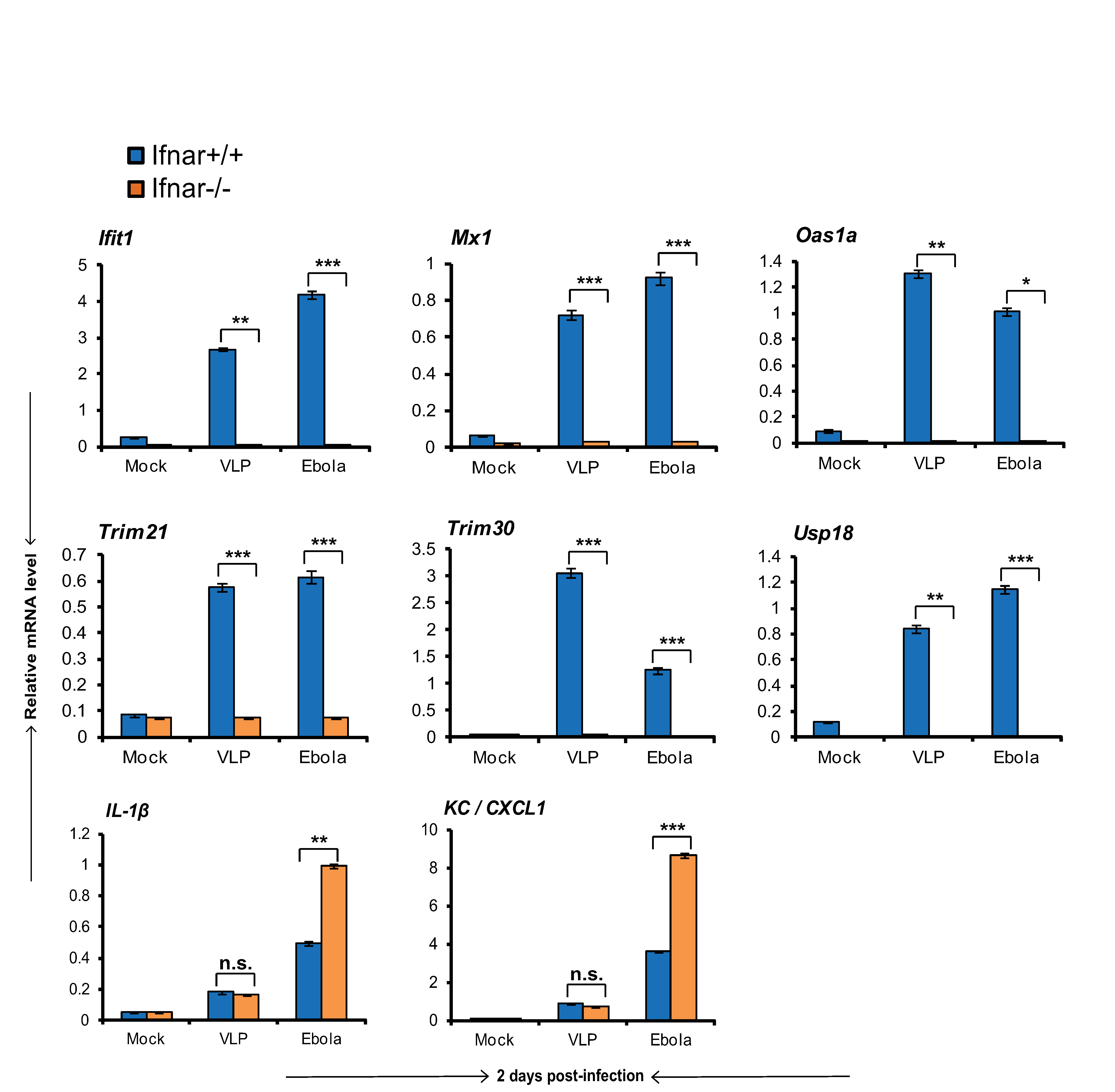

Supplement: S2 Fig — Ifnar +/+ and Ifnar -/- mice (n = 5/group) were injected with VLP alone and expression of indicated ISGs and cytokine/chemokine in liver was measured on day 2 as shown in Fig. 3. Ebola infected mice served as positive control. Data represent the mean of duplicate samples from more than three independent experiments ± SEM. *** denotes P ≤ 0.001, ** P ≤ 0.01, *P ≤ 0.05. n.s., No significance. (TIF) [file pone.0118345.s002.tif]

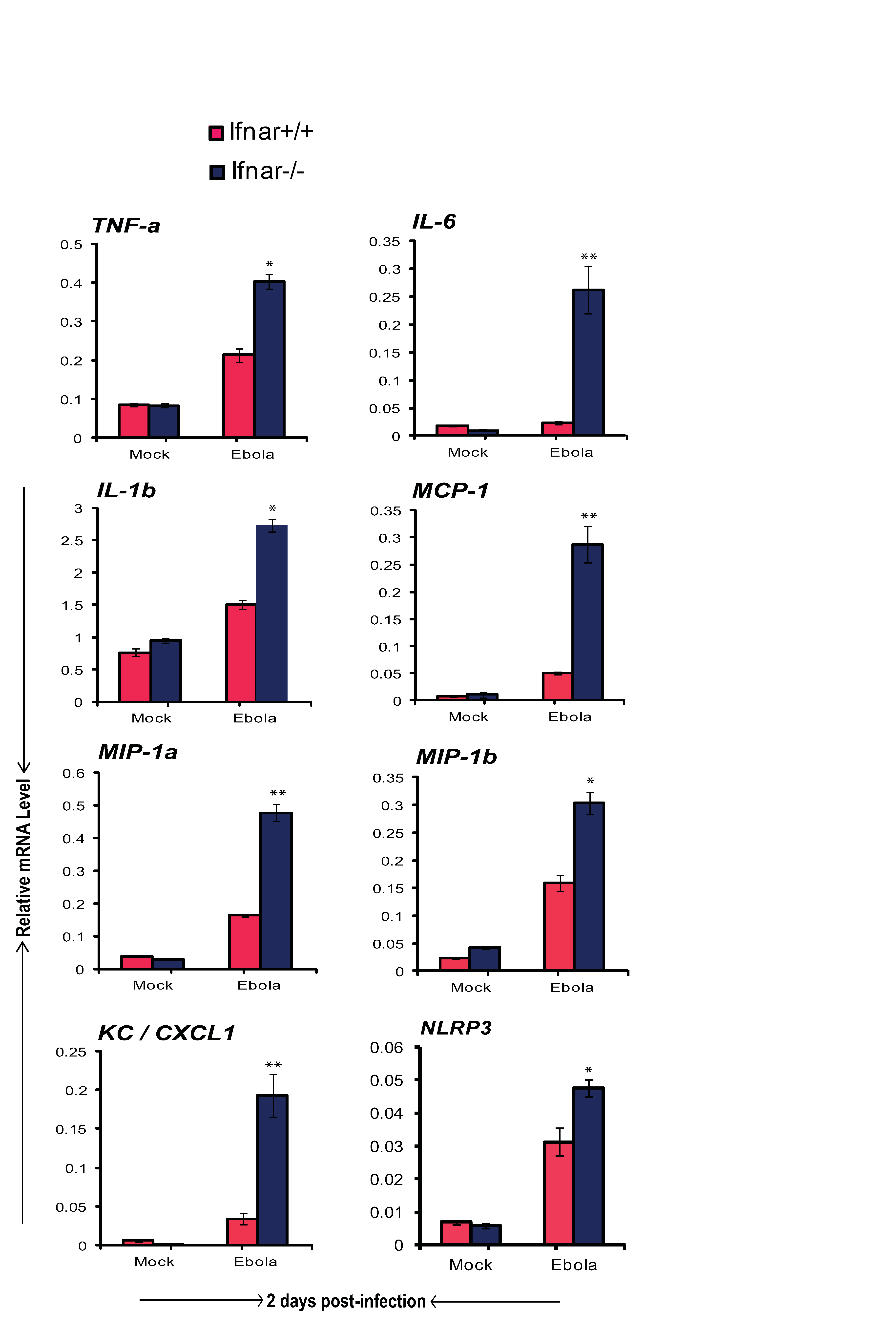

Supplement: S3 Fig — RNA from spleen of Ifnar +/+ and Ifnar -/- mice (n = 5/group) infected with EBOV was tested for expression of indicated cytokines or chemokines on day 2 post-infection. Data represent the mean of duplicate samples from three individual experiments ± SEM. Asterisks denote **P ≤ 0.01 and *P ≤ 0.05. (TIF) [file pone.0118345.s003.tif]

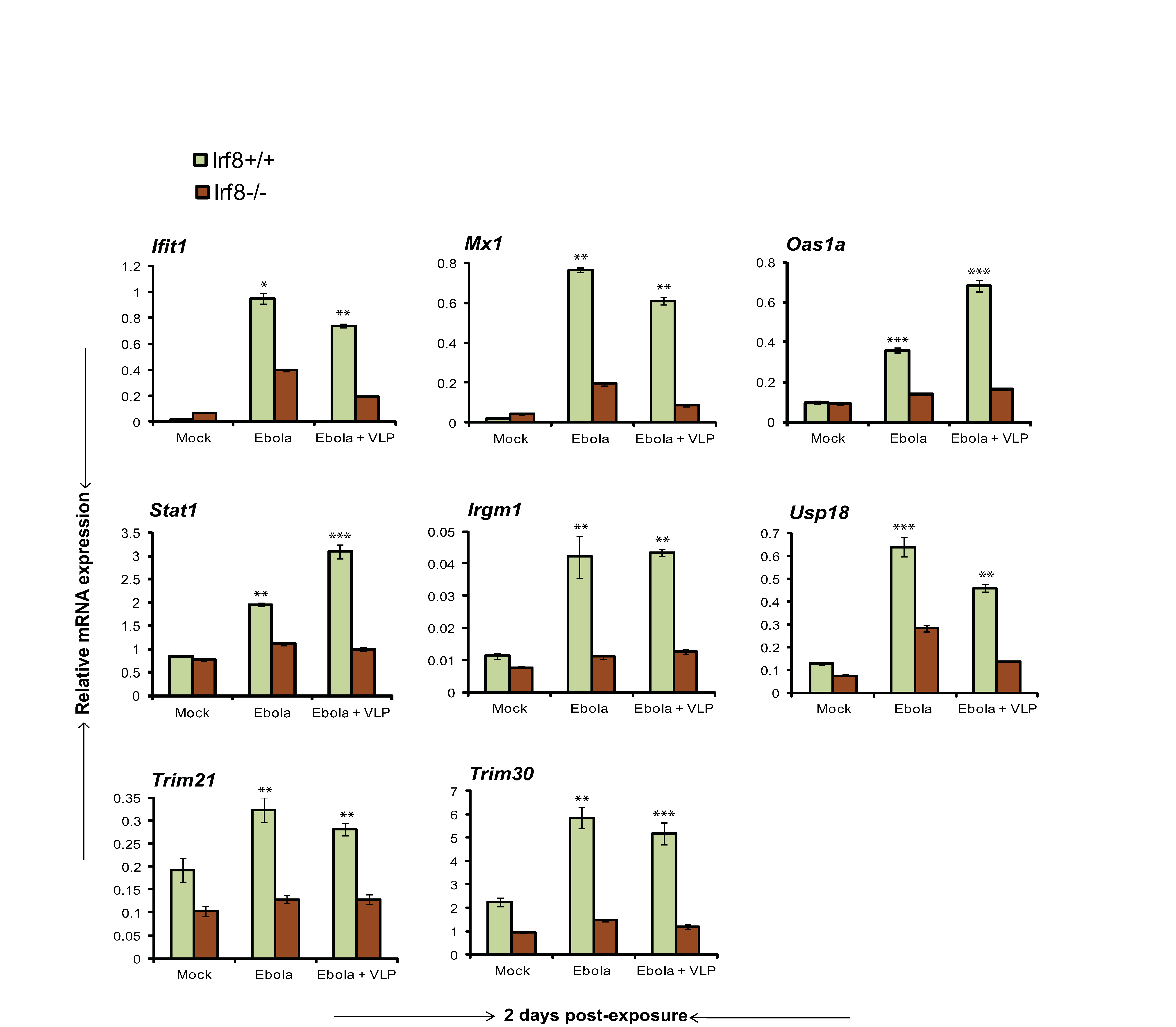

Supplement: S4 Fig — Relative mRNA expression of indicated ISGs in spleen on day 2 post-infection (n = 5/group) was analyzed as mentioned in Fig. 6C. Data are the mean of duplicate samples ± SEM and a representative of three independent experiments. *** indicates P ≤ 0.001, ** P ≤ 0.01 and *P ≤ 0.05. (TIF) [file pone.0118345.s004.tif]
